# Activation of Th lymphocytes alters pattern expression and cellular location of VIP receptors in healthy donors and early arthritis patients

**DOI:** 10.1038/s41598-019-43717-2

**Published:** 2019-05-14

**Authors:** R. Villanueva-Romero, I. Gutiérrez-Cañas, M. Carrión, I. González-Álvaro, J. M. Rodríguez-Frade, M. Mellado, C. Martínez, R. P. Gomariz, Y. Juarranz

**Affiliations:** 1 Departamento de Biología Celular, Universidad Complutense de Madrid, Instituto de Investigación Sanitaria Hospital 12 de Octubre (imas12), Madrid, Spain; 2 Servicio de Reumatología, Instituto de Investigación Sanitaria Hospital La Princesa (IIS-IP), Madrid, Spain; 3 Departamento de Inmunología y Oncología, Centro Nacional de Biotecnología, Consejo Superior de Investigaciones Científicas, Madrid, Spain

**Keywords:** CD4-positive T cells, Neuroimmunology

## Abstract

Vasoactive Intestinal Peptide (VIP) is an important immunomodulator of CD4^+^ cells in normal and pathological conditions, which exerts its anti-inflammatory and immunomodulatory actions through VPAC receptors, VPAC_1_ and VPAC_2_. Only a decrease in the expression of VPAC_1_ mRNA on Th cells upon activation has been reported. Thus, the deepening in the knowledge of the behavior of these receptors may contribute to the design of new therapies based on their activation and/or blockade. In this study, we describe the expression pattern, cellular location and functional role of VIP receptors during the activation of human Th cells in healthy conditions and in early arthritis (EA). The protein expression pattern of VPAC_1_ did not change with the activation of Th lymphocytes, whereas VPAC_2_ was up-regulated. In resting cells, VPAC_1_ was located on the plasma membrane and nucleus, whereas it only appeared in the nucleus in activated cells. VPAC_2_ was always found in plasma membrane location. VIP receptors signaled through a PKA-dependent pathway in both conditions, and also by a PKA-independent pathway in activated cells. Both receptors exhibit a potent immunomodulatory capacity by controlling the pathogenic profile and the activation markers of Th cells. These results highlight a novel translational view in inflammatory/autoimmune diseases.

## Introduction

CD4^+^ T helper (Th) cells are decisive in the struggle against pathogens and in the maintenance of immune homeostasis. The activation and differentiation of Th cells can take many forms and generate immune memory. Anyhow, the extracellular environment has particular importance in the control of these processes both in homeostatic and pathological conditions. Over the last few years, several studies have shed light on the important role of neuroendocrine milieu in both lymphoid cells activation and differentiation. In this sense, vasoactive intestinal peptide (VIP) is one of the best studied neuropeptides that exerts a wide spectrum of actions on the immune system, from innate to adaptive immunity. Overall, VIP can be considered as an anti-inflammatory and immunomodulatory agent^1–3^. VIP modulates the differentiation of the different subsets of Th cells, increasing Th2 and T regulatory (Treg) subsets and decreasing Th1 and pathogenic Th17 cells^1,2,4–9^. This neuropeptide also regulates the heterogeneity and plasticity of Th17 cells in physiological and pathological conditions^9–11^. Moreover, healing effects of VIP in animal models of inflammatory/autoimmune diseases have been reported^1–5^. VIP is able to modulate all the stages comprised between the arrival of pathogens and Th cell differentiation in rheumatoid arthritis (RA) through its known anti-inflammatory and immunomodulatory actions^6,10–15^. Furthermore, early RA (eRA) or early spondyloarthritis patients with low VIP serum levels present a worse clinical course despite receiving more intense treatment^16,17^.

VIP exerts its actions through its specific receptors, VPAC_1_ and VPAC_2_^18^. Th lymphocytes from mouse and human express both receptors^1,19–21^. Their mRNA expression pattern in Th cells changes in human pathologies, such as eRA or HIV infection^9–11,22^. Human and mouse *in vitro* T cell activation results in a loss of VPAC_1_ mRNA expression^20,21^. Moreover, VPAC_1_ might be useful as an activity marker since eRA patients with more severe inflammation and higher disease activity show lower levels of this receptor^23^. VPAC_2_ mRNA is induced after *in vitro* activation of mouse lymphocytes^19^, whereas low levels of VPAC_2_ have been described in resting human Th cells^20^.

The deepening in the knowledge of the behavior of these receptors may contribute to the design of new therapies based on their activation and/or blockade. Therefore, the aim of the present work is the study of protein/mRNA expression pattern, cellular location, signaling pathways and functionality of VIP receptors during the activation of human memory Th cells in healthy conditions and in one rheumatic pathology, EA. The understanding of protein expression and localization of these receptors can give the scientific community more significant information than the mRNA expression known so far. Moreover, changes in their localization and signaling pathway following the activation of the cells can arise in different therapeutic approaches.

## Results

### The expression pattern and cellular location of VPAC receptors change with the activation of human memory Th lymphocytes from healthy donors

**Figure 1:**
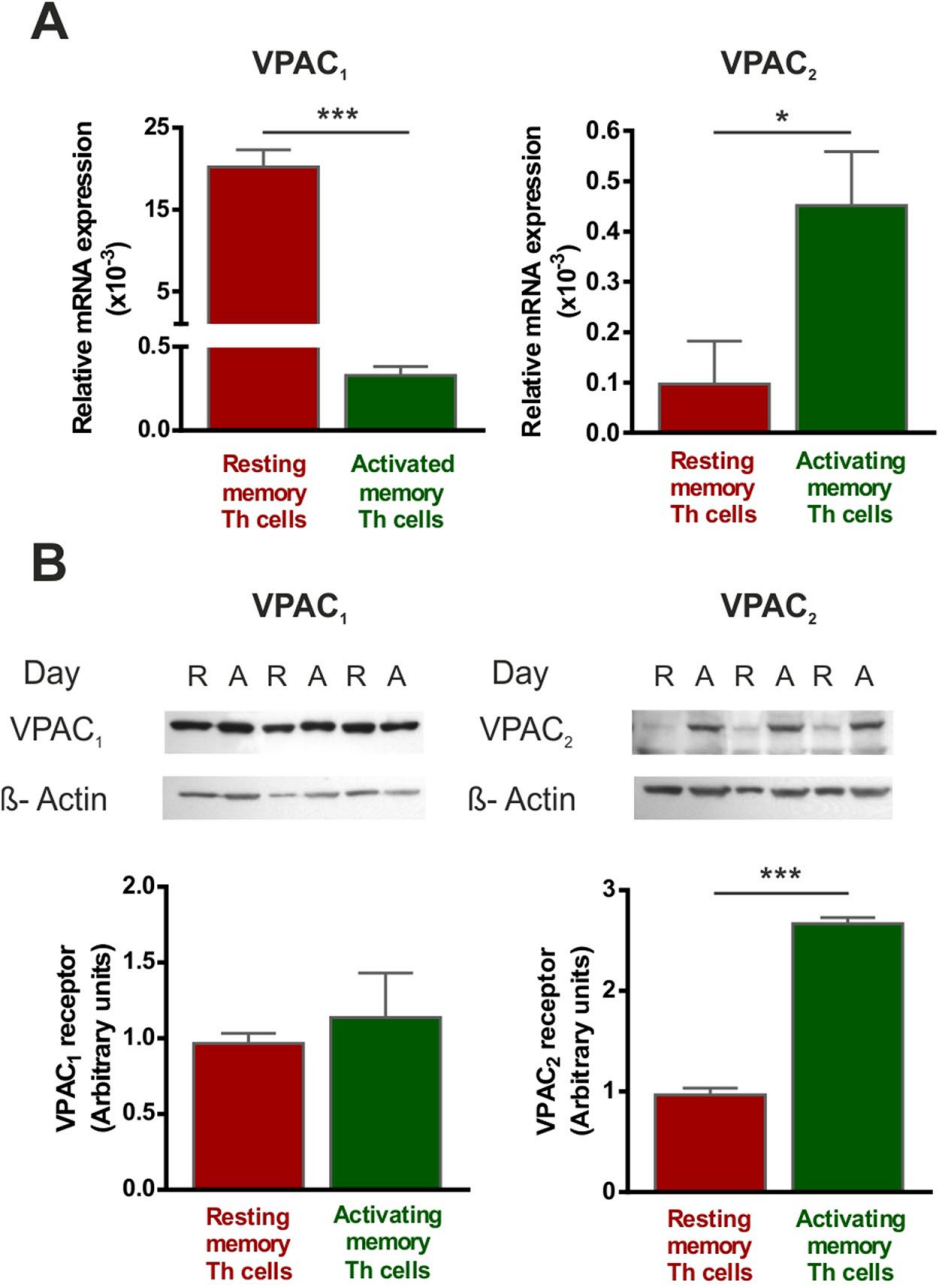
The expression pattern of VPAC receptors changes with the activation of memory Th lymphocytes from HD. (**A**) mRNA expression of VPAC_1_ and VPAC_2_ receptors was determined by semiquantitative real-time PCR analysis in resting- and seven days activated- Th cells. Results are expressed as relative mRNA levels (normalized to ACTB mRNA levels, 2^−∆Ct^). The mean ± SEM of triplicate determination of seven different HD samples are shown (*p < 0.05, ***p < 0.001). (**B**) Protein levels of VPAC_1_ and VPAC_2_ receptors in lysates of resting- and seven days activated- memory Th cells were measured by Western blotting. β-actin protein levels were determined as a loading control. Protein bands were analyzed by densitometric analysis and normalized against the intensity of β-actin. Results represent the mean ± SEM of seven different donors (***p < 0.001).

**Figure 2:**
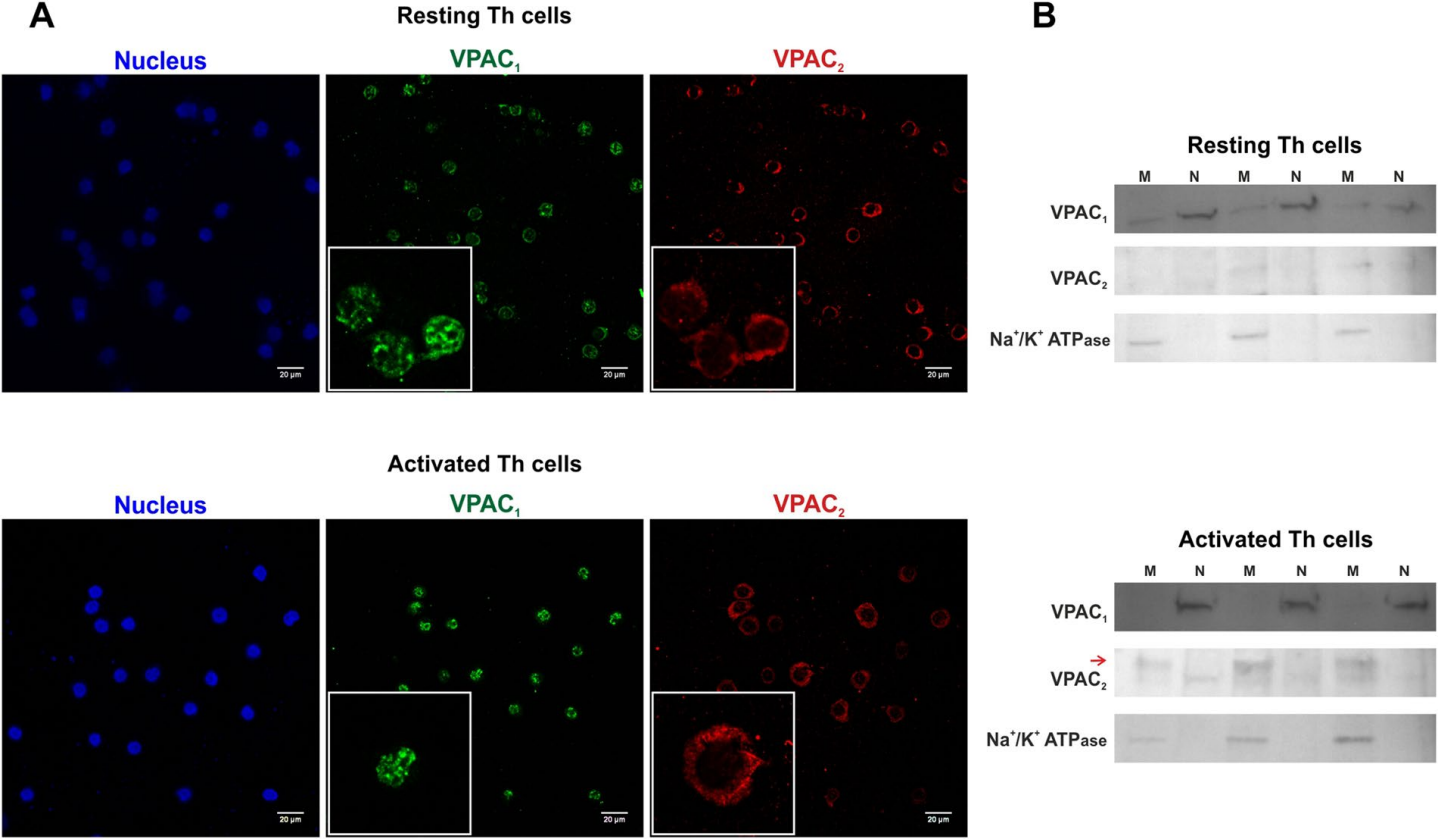
Cellular localization of VPAC receptors during the activation of memory Th lymphocytes from HD. (**A**) Immunofluorescence analysis on resting- (upper) and seven days activated- Th cells (lower) using specific antibodies for VPAC_1_ (Alexa Fluor 488, green), and VPAC_2_ (Alexa Fluor 594, red). Nuclei were counterstained with Hoechst (Blue). Fluorescence was examined on Leica SP2 AOBS confocal microscope (63X). Original scale bars, 20 µm. Boxed areas show higher magnification views of individual cells (3,17x zoom). Results are representative of five different donors. (**B**) VPAC_1_ and VPAC_2_ receptors in plasma membranes and nuclear fraction of resting- and seven days activated- memory Th cells were measured by Western blotting. Na^+^/K^+^ ATPase protein levels were determined as plasma membrane marker. Three of five different donors were shown.

**Figure 3:**
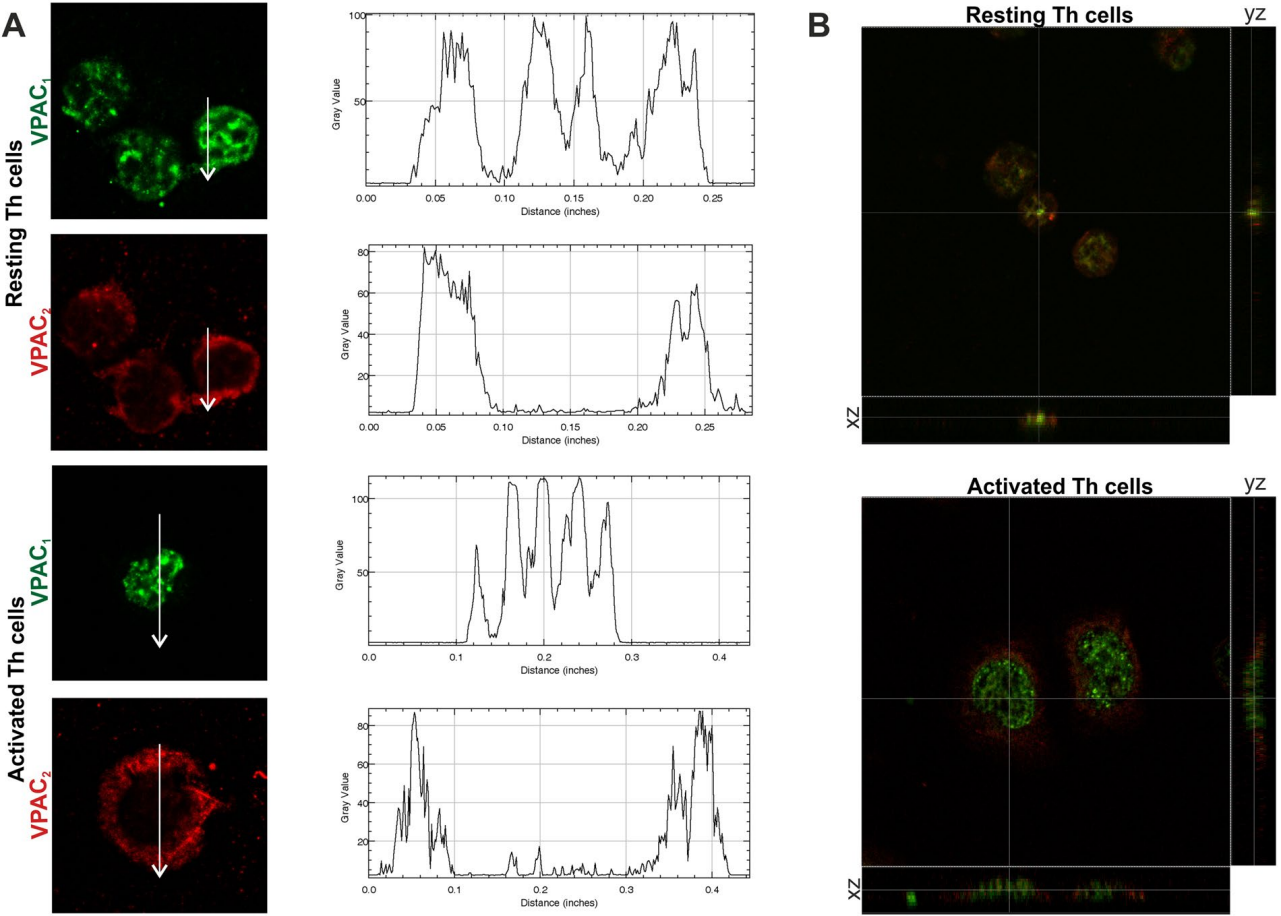
Distribution analysis of VPAC receptors across the Th lymphocytes from HD. Zoomed 63x images were imported into ImageJ to analyze the distribution of VIP receptors across the cell. (**A**) Representative graphs of intensity profiles plotted as a function of distance (measured in pixels) versus intensity (measured in gray-scale values) showed a distinctive distribution of receptors on each cell states. (**B**) Orthogonal projections of merged images from resting (up) and activated cells (down). In the lower side: XZ plane; in the right side: YZ plane.

To determine whether the activation of human memory Th lymphocytes induces changes in the expression pattern of VPAC receptors, we measured mRNA levels of both receptors by semiquantitative real-time RT-PCR (Fig. 1A). After seven days of activation/expansion, memory Th lymphocytes showed decreased VPAC_1_ but increased VPAC_2_ mRNA expression. These changes were observed even after 24 h of activation (Table 1). Protein expression of VIP receptors was analyzed by western-blot (WB) and immunofluorescence staining. WB studies indicated that VPAC_2_ protein expression is higher in activated memory Th cells, however no differences were found in VPAC_1_ protein expression between resting and activated Th cells (Fig. 1B). These data indicate that changes in VPAC_1_ receptor transcripts were not found at protein level. The immunofluorescence staining studies corroborated the presence of both VPAC receptors in resting and activated memory human Th cells, however, it should be noted that intracellular location of both receptors showed a different pattern (Fig. 2A). In resting Th cells, VPAC_1_ receptor seems to appear in plasma membrane and nuclear regions, whereas in activated Th cells it is only found in nuclear location. To support this idea, we performed the subcellular fractionation into nuclear region and plasma membrane, and subsequent WB analysis (Fig. 2B). VPAC_2_ receptor appeared only in plasma membrane location in both resting- and activated- Th cells, although the presence of this receptor was greater in activated than resting cells. To verify, we performed a distribution analysis by fluorescence intensity and 3D vision which confirms our previous thought (Fig. 3).

**Table 1:** Time-course expression of VPAC receptors during the activation of memory Th cells from healthy donors.

Activation/expansion days	VPAC_1_ receptor	VPAC_2_ receptor
0 (Resting cells)	20.1 ± 2.09	0.11 ± 0.09
1	1.13 ± 0.14***	0.42 ± 0.08*
4	0.26 ± 0.04***	0.40 ± 0.16
7	0.33 ± 0.54***	0.45 ± 0.11*

mRNA expression of VPAC_1_ and VPAC_2_ receptors was determined by real-time PCR analysis in resting and after 1, 4 or 7 days of activation/expansion. Results are expressed as relative mRNA levels (relative to ACTB mRNA levels, 2^−∆Ct^). The mean ± SEM of triplicate determination of five independent experiments are shown (*p < 0.05, ***p < 0.001). Relative mRNA expression (x 10^−3^).

**Figure 4:**
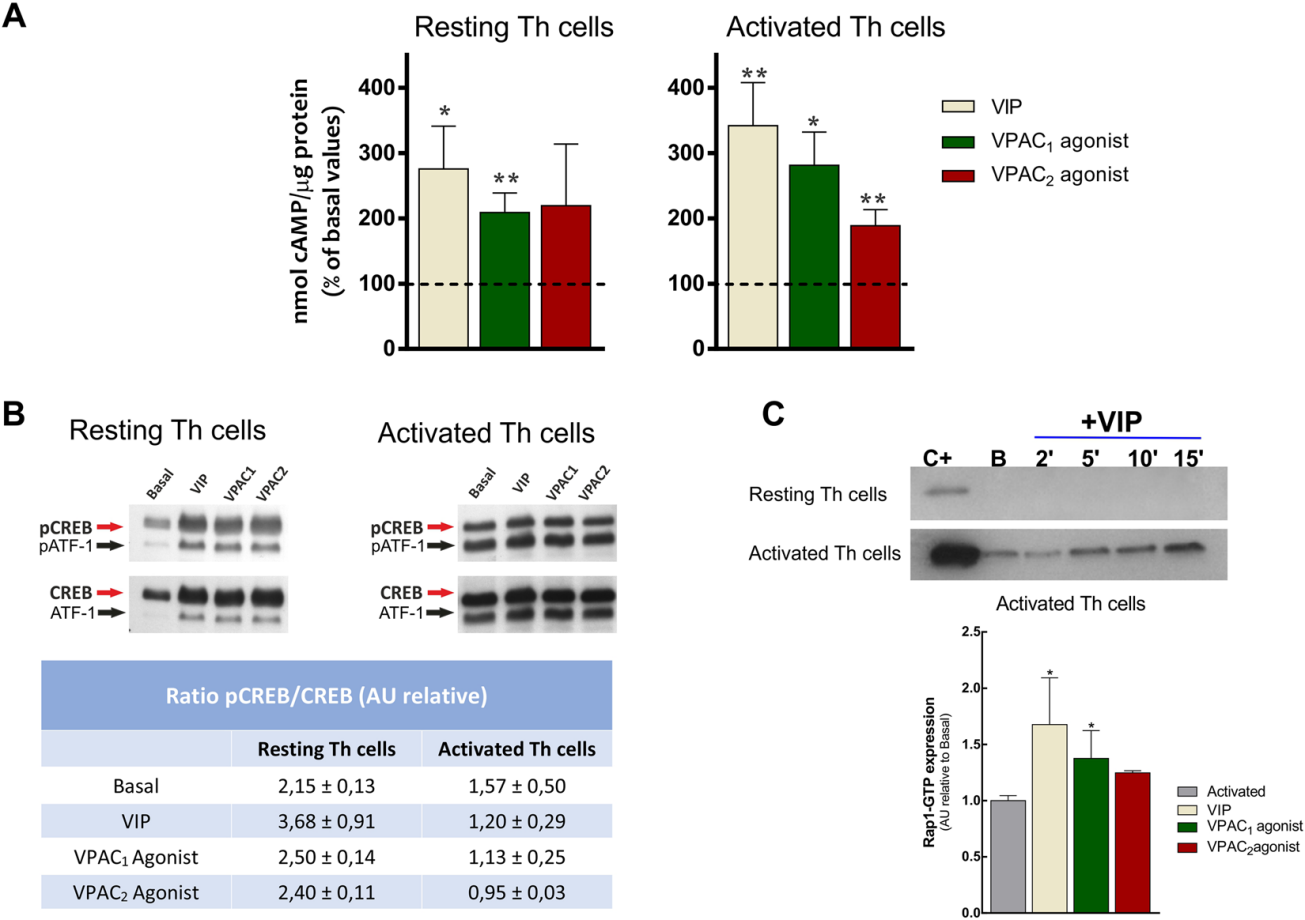
VPAC receptors signal through canonical and non-canonical pathways in memory Th lymphocytes from HD. (**A**) Intracellular levels of cAMP under basal conditions and after 1 h treatment with 10 nM of VIP, VPAC_1_ agonist or VPAC_2_ agonist was determined in resting- and seven days activated- Th cells by ELISA analysis. Data are the mean ± SEM of duplicate determination of five different HD samples (*p < 0.05, **p < 0.01). Dashed lines represent the basal condition. (**B**) Western blot analysis of CREB and phosphorylated CREB (p-CREB) under basal conditions and after 1 h treatment with 10 nM of VIP, VPAC_1_ agonist or VPAC_2_ agonist was determined in resting- and seven days activated- Th cells. Activating transcription factor-1 (p-ATF1), the other CREB family member, is also shown. Table show the ratio p-CREB/CREB of relative densitometry units. Values are the mean ± SEM of relative densitometry units for each band of five different donors. (**C**) Up*per: R*esting- and seven days activated- Th cells were treated with VIP for different times. Rap1 activation was measured by GST-RSD pulldown followed by western blot with anti-Rap1 antibody. Positive control was obtained by treatment of lysates with GTPγS. A representative experiment of five other is shown. *Down*: Seven days activated Th lymphocytes were treated with VIP, VPAC_1_ agonist and VPAC_2_ agonist during 15 min. Rap1 activation was measured by GST-RSD pulldown followed by western blot with anti-Rap1 antibody. Data are the mean ± SEM of duplicate determination of five different donors (*p < 0.05).

To check the functionality of these receptors in resting- and activated- Th cells we studied the signaling pathways triggered by cAMP, the main second messenger induced by both receptors. VIP, VPAC_1_ agonist and VPAC_2_ agonist increased cAMP levels in resting- and activated- Th cells (Fig. 4A). Next, we tested the specific pathways downstream this second messenger, namely PKA-dependent or canonical pathway and PKA-independent or non-canonical pathway. As the phosphorylation of transcription factor CREB is downstream PKA signalling, we measured it to assess the PKA-dependent pathway. VPAC agonists increased the ratio pCREB/CREB mainly in resting cells (Fig. 4B). Besides, the GTPase Rap1 is downstream of cAMP-activated guanine nucleotide exchange factor (EPAC), mediator of PKA-independent pathway. The presence of Rap1-GTP was only detected in activated Th cells after VIP treatment, gradually increasing from 2 min to 15 min of treatment (Fig. 4C, *upper*). After 15 min of stimulation, VIP and the two VPAC agonists induced the activation of Rap1 (Fig. 4C, *down*). Therefore, VPAC receptors mainly signal through the PKA-dependent pathway in resting Th cells, whereas the PKA-independent pathway is triggered in activated Th cells.

### Involvement of VPAC receptors in the immunomodulatory role of VIP during the activation of human memory Th cells from healthy donors

**Figure 5:**
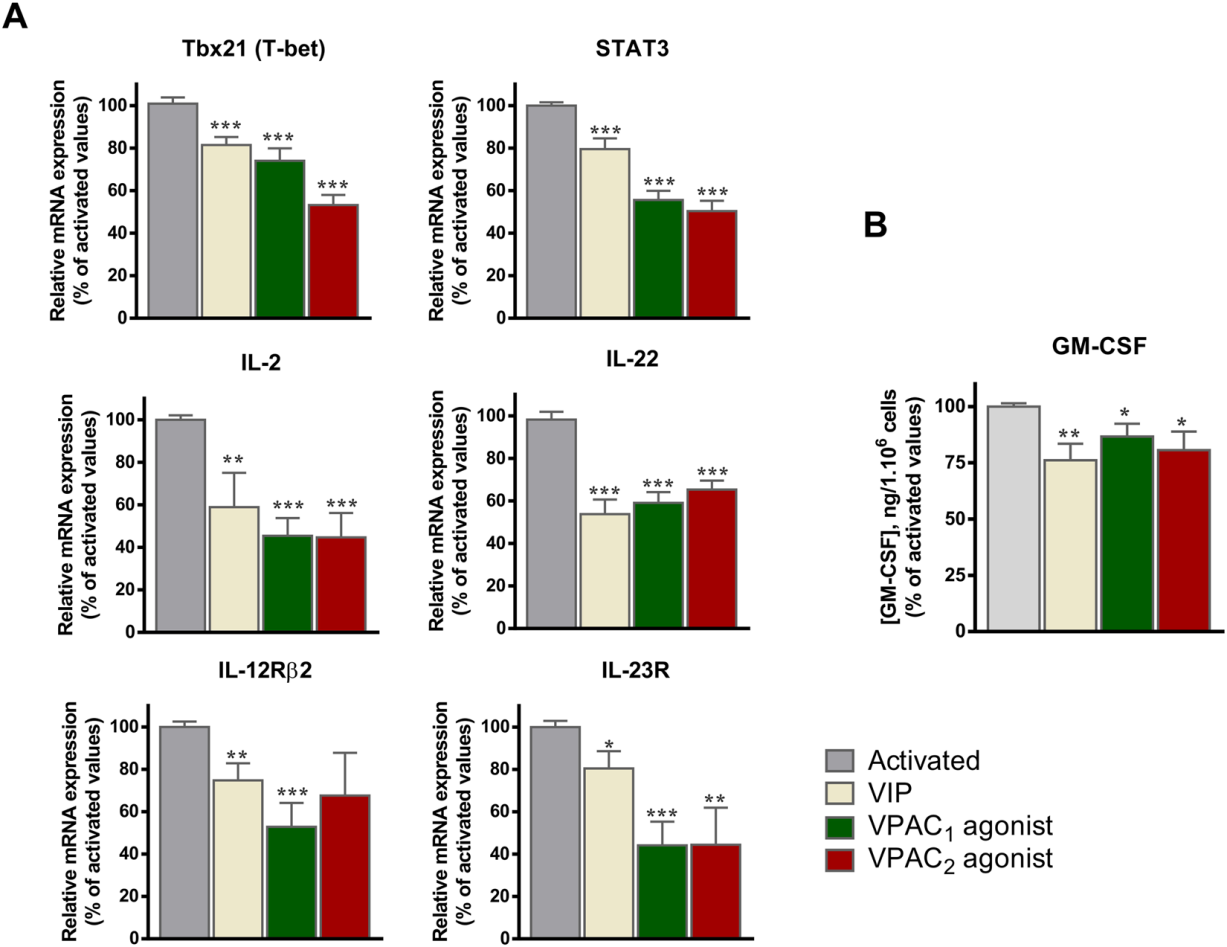
VPAC_1_ and VPAC_2_ receptors are involved in the immunomodulatory role of VIP during the activation of human memory Th cells from healthy donors. (**A**) mRNA expression of the transcription factors (STAT3 and Tbx21), cytokines (IL-2 and IL-22) and receptor cytokines (IL-12Rβ2 and IL-23R) was determined by real-time PCR analysis in resting- and one day activated- Th cells. Data were normalized with ACTB mRNA expression and are shown as the percentage of activated Th cells values. The mean ± SEM of triplicate determination of five different HD samples are shown (*p < 0.05, **p < 0.01, ***p < 0.001). (**B**) Protein expression of GM-CSF was analysed in culture supernatants by ELISA. Data are the mean ± SEM of duplicate determination of five different HD samples (*p < 0.05, **p < 0.01).

VIP modulates several molecules associated with a pathogenic profile during the activation of human memory Th cells^10^. Thus, we next determined the VPAC receptor subtype involved in this action, checking mRNA or protein expression of cytokines (IL-2, IL-22, GM-CSF), cytokine receptors (IL-12Rβ2, IL-23R) and transcription factors (STAT3, T-bet) associated with a pathogenic profile in these cells. Time-course response during the activation/expansion of cells showed that all molecules peaked at 1 day of activation (data not shown). As change in VPAC receptor expression is induced after 1 day of cell activation (Table 2), we tested the effect of VIP as well as VPAC agonists on these molecules (Fig. 5). Both VPAC_1_ and VPAC_2_ receptors modulated the pathogenic profile of memory Th cells after 1 day of activation, decreasing the expression of transcription factors, cytokines and cytokine receptors related to the pathogenic profile of memory Th cells.

**Table 2:** Genes analyzed by semiquantitative real-time polymerase chain reaction.

Gene	GeneBank accession no.	Sequence position/assay location (TaqMan®)	Sequence/assay ID (TaqMan®)
IL-22	NM_020525.4	445	Hs01574154_m1
IL-2	NM_000586.3	267	Hs00174114_m1
Tbx21 (T-bet)	NM_013351.1	707	Hs00203436_m1
STAT3	NM_003150.3	488	Hs01047580_m1
IL-12Rβ2	NM_001559.2	1,900	Hs00155486_m1
IL-23R	NM_144701.2	1,037	Hs00332759_m1
VPAC_1_	NM_004624.3	306	Hs00270351_m1
VPAC_2_	NM_003382.4	644	Hs00173643_m1
β-Actin	NM_001101.3	54	Hs03023943_g1

Gene, genebank accession number, sequence position or assay location, and sequence or assay ID for each primer used in the study are shown.

### Contribution of VPAC receptors in the activation/proliferation/chemotaxis of human memory Th cells from healthy donors

**Figure 6:**
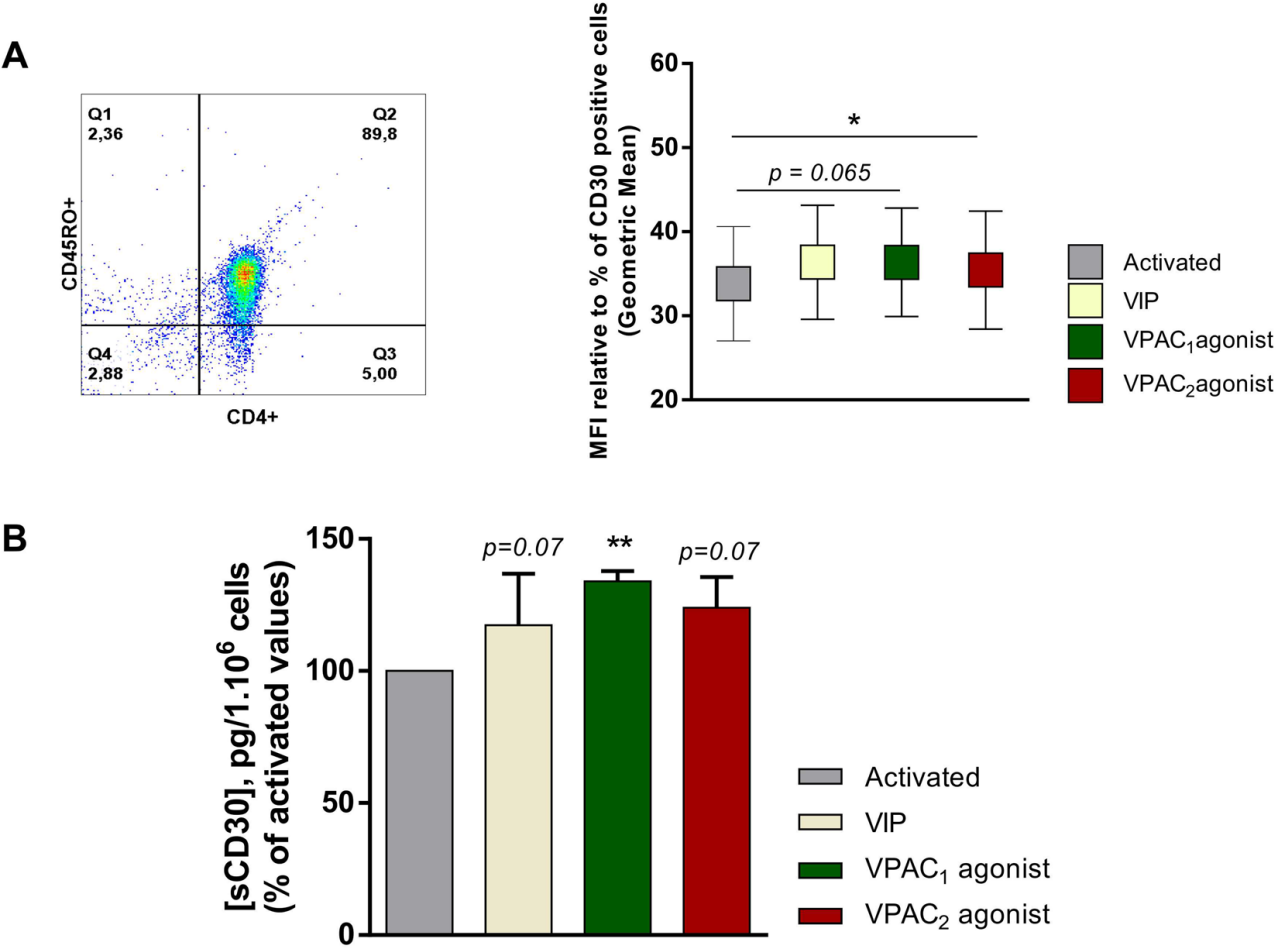
CD30 expression is increased by VIP by both receptors in activated memory Th cells from healthy donors. (**A**) Expression of CD30 was determined by flow cytometry in activated Th cells at day 4. Auto-fluorescence and isotype controls were set up to determine the non-specific fluorescence signal and percentage of total CD30 positive cells was quantified. The indicated proportion of positive cells was determined in the gate of CD4^+^CD45RO^+^ cells (a representative dot plot is shown). Comparison of CD30 Geometric Mean fluorescence intensity (gMFI) in the different conditions is shown. The values of gMFI was corrected by the percentage of CD30^+^ cells in each condition. Data are the mean ± SEM of five different donors performed by duplicated (*p < 0.05). (**B**) Soluble CD30 was analysed in culture supernatants by ELISA in activated Th cells at day 4. Results are the mean ± SEM of duplicate determinations of six different HD samples (**p < 0.01).

**Figure 7:**
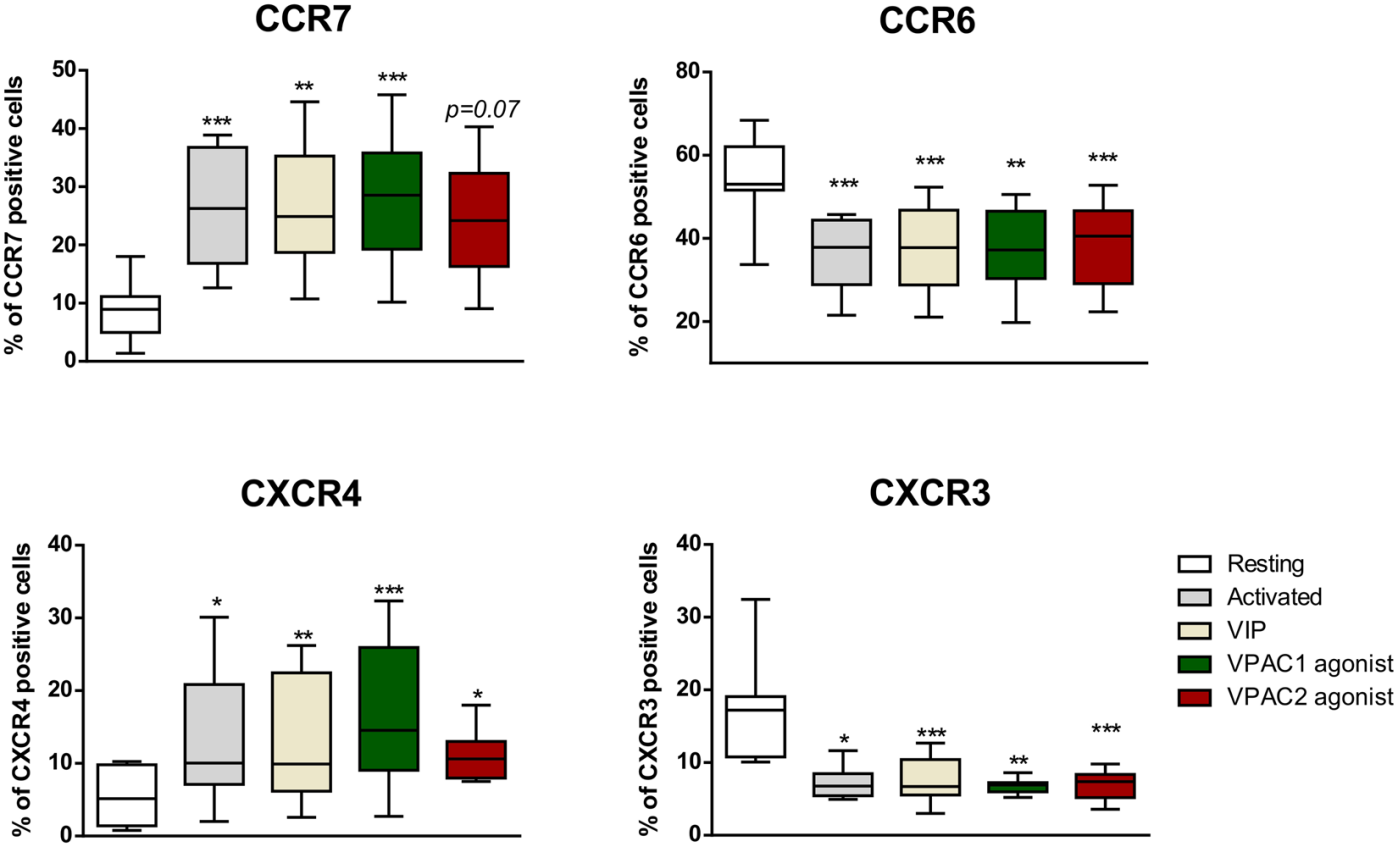
VIP and its receptors maintain the migration capacity of activated memory Th cells from healthy donors. Expression of CCR6, CCR7, CXCR3 and CXCR4 was determined by flow cytometry in resting and one day activated Th cells. Auto-fluorescence and isotype controls were set up to determine the non-specific fluorescence signal and percentage of total positive cells was quantified. Results represent the mean ± SEM of duplicate determinations of five different HD samples (*p < 0.05, **p < 0.01, ***p < 0.001).

To study the involvement of each VPAC receptor in the activation of Th cells, we tested CD69, CD25, CD62L, CD154 and CD30 as activation markers. During the activation, the highest expression levels for all these molecules was at day 1, except for CD30 that was at day 4 (data not shown). No changes were observed in the presence of VIP or the VPAC agonists in the number of CD69^+^, CD25^+^, CD62L^+^ or CD154^+^ cells during the activation (data not shown); however, VIP increased the percentage of CD30^+^ cells and the secretion of this activation marker, probably signaling through both receptors (Fig. 6). Then, we decided to check the effect of each receptor subtype in the cell growth/survival during cell activation/expansion. No changes were observed in cell number in the presence of VIP, VPAC_1_ agonist or VPAC_2_ agonist (data not shown). To test the migration capacity, we studied the expression of several chemokine receptors. After one day of activation, Th cells increased the CCR7 and CXCR4 percentage of positive cells with respect to resting cells, whereas the number of CCR6- and CXCR3-positive cells was diminished (Fig. 7). To assess whether VIP or its receptors alter the functionality of these chemokines receptors during the activation, we performed a cell migration assay in the presence of suboptimal concentrations of their specific ligands, CCL19, CXL12, CCL20 and CXCL10, respectively in resting and one day activated Th cells. Th cells activation increased cell migration in response to CCL19, CXCL10 and CXCL12, but not to CCL20, a chemokine for CCR6 receptor. The presence of VIP during the activation maintained the capacity of migration of these cells to all chemokine tested (data not shown). In summary, VIP and its receptors increased the expression of CD30 and maintained the chemotactic capacity of Th cells during their activation.

### Comparative studies in human memory Th cells from EA patients: pattern expression and cellular localization of VIP receptors

**Figure 8:**
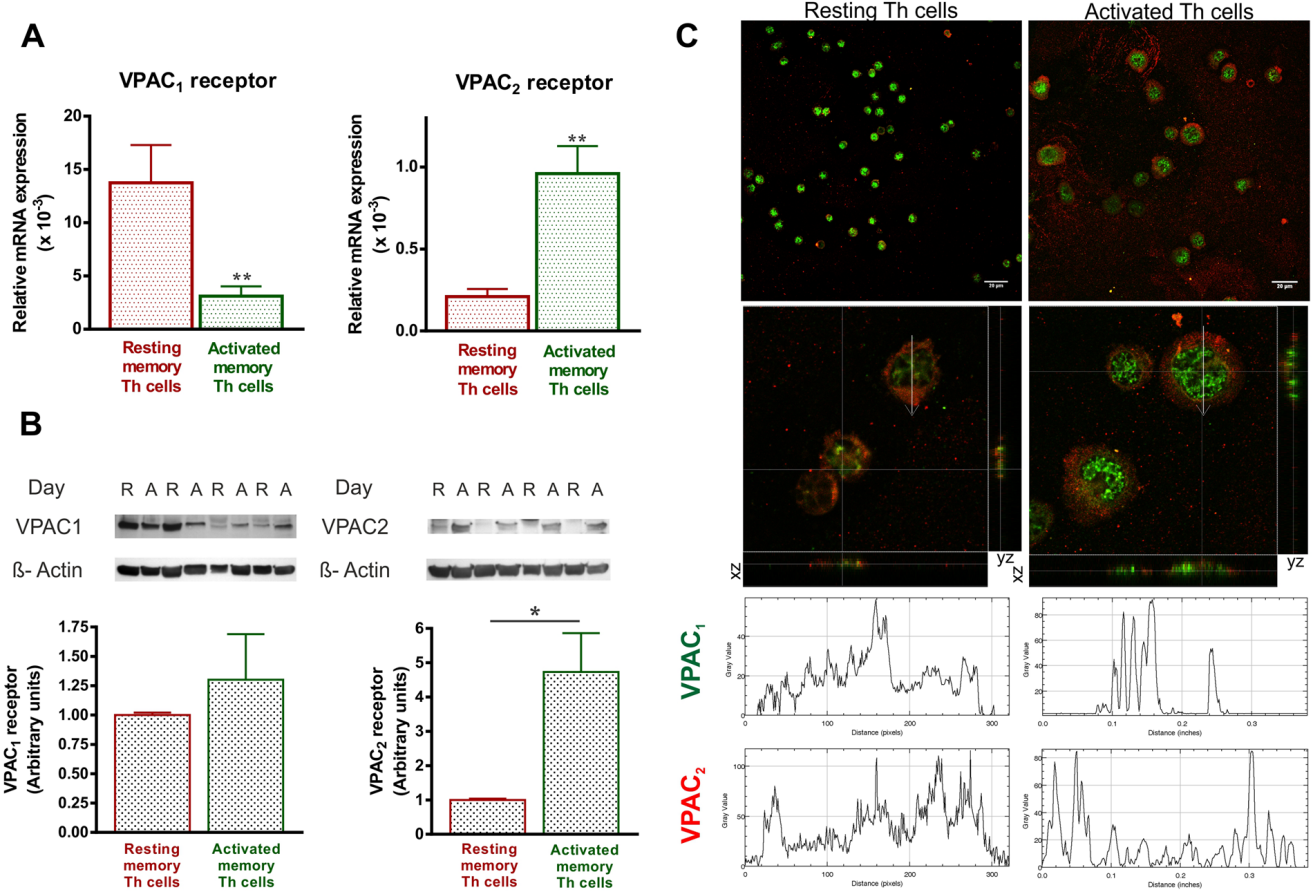
Changes in the expression pattern and cellular localization of VPAC receptors in EA patients. (**A**) mRNA expression of VPAC_1_ and VPAC_2_ receptors was determined by real-time PCR analysis in resting and seven days activated Th cells from EA patients. Results are expressed as relative mRNA levels (normalized to ACTB mRNA levels, 2^−∆Ct^). The mean ± SEM of triplicate determination of seven EA patients are shown (**p < 0.01). (**B**) Protein levels of VPAC_1_ and VPAC_2_ receptors in lysates of resting and seven days activated memory Th cells from EA patients were measured by Western blotting. β-actin protein levels were determined as a loading control. Protein bands were analyzed by densitometric analysis and normalized against the intensity of β-actin. Results represent the mean ± SEM of five different EA patients (*p < 0.05). (**C**) Immunofluorescence analysis on resting (left side) and seven days activated Th cells (right side) using specific antibodies for VPAC_1_ (Alexa Fluor 488, green), and VPAC_2_ receptors (Alexa Fluor 594, red). *Upper*: Fluorescence was examined on Leica SP2 AOBS confocal microscope (63X). Original scale bars, 20 µm. *Medium*: Orthogonal projections of magnified 63x images (3,17x zoom). In the lower side: XZ plane; in the right side: YZ plane. *Lower*: Representative graphs of intensity profiles plotted as a function of distance (measured in pixels) versus intensity (measured in gray-scale values). Results are representative of five different EA patients.

To investigate if the observed changes in the expression pattern of VPAC receptors during the activation of memory Th cells also occur in pathological conditions, we tested their expression in memory Th lymphocytes from EA patients (Fig. 8). Transcripts of VPAC_1_ receptor decreased after seven days of activation; nevertheless, an increase of VPAC_2_ receptor mRNA expression was detected. Indeed, VPAC_2_/VPAC_1_ ratio was increased with the activation of Th lymphocytes from EA patients compared to resting cells (0.31 ± 0.05 and 0.01 ± 0.002, respectively). Regarding protein expression, we found no changes in VPAC_1_ expression and an upregulation of VPAC_2_ receptor. Distribution analysis by fluorescence intensity and 3D vision shown that VPAC_1_ appeared both in plasma membrane and nuclear localization in resting cells whereas after activation of Th cells from EA patients only nuclear location was found. Cellular location of VPAC_2_ receptor is limited to plasma membrane in both cell conditions.

## Discussion

The immunomodulatory role of VIP during the activation of Th cells has been clearly described in both healthy and pathological conditions, controlling their activation, polarization and plasticity^1,6–11^. Nevertheless, the implication of each VPAC receptor subtype has not yet been elucidated, especially taking into account previous studies indicating a change in the expression pattern of VPAC receptors in these cells^20,21^. The present study shows changes in the expression pattern, the cellular localization and the functional outcomes of these receptors during the activation of human Th cells. It is important to describe the first two items before trying to solve and understand the last one.

Concerning the expression pattern of VPAC_1_ in resting and activated memory Th cells from healthy donors (HD), we corroborate the down-regulation of VPAC_1_ mRNA expression with cell activation, previously described in cells of the immune network^11,20,21^. It has been demonstrated that the transcription factor Ikaros sets the threshold for TCR activation and undergoes an increase during T cell activation, engaging the VPAC_1_ promotor at Ikaros binding motifs and directly suppressing the transcription of VPAC_1_ gene^24^. As far as we know, this is the first study which reports the protein expression pattern of VPAC_1_ receptor during the activation of Th cells. Our data indicate that VPAC_1_ protein does not change with cells activation which may be caused by an increase in the mRNA stability, by a rise in protein translation rate, or by a decrease in the receptor destruction in the lysosomes, during the activation of Th cells.

Regarding the expression pattern of VPAC_2_, it has only been described that it is expressed at very low levels in human resting T cells, whereas in mouse T lymphocytes is detected only following stimulation with CD3^19,20^. Our studies confirm that VPAC_2_ expression is very low in resting cells, but indicate a significant upregulation after Th activation in HD, both at mRNA and protein level, pointing out that VPAC_2_ is an inducible receptor during the activation of human Th cells. The overexpression of this receptor is not only related to lymphocytes activation, but also to their differentiation. In this regard, it has been described that the VIP/VPAC_2_ axis mediates the Th2 differentiation in mouse Th cells and the increase in VPAC_2_ expression after Th17 polarization of human naïve Th cells^9,25^.

When we compared the expression pattern of VPAC receptors during the activation of Th lymphocytes from healthy donors with that of EA patients, we found similar results at protein level, with unchanged VPAC_1_ expression and up-regulation of VPAC_2_ receptor. Data related to both VPAC transcripts were different to protein expression, probably due to a post-transcriptional regulation. In this sense, we observed a loss in mRNA expression of VPAC_1_ receptor, similar to data from HD, although to a lesser extent and starting with lower expression. These results are in agreement with our previous results which indicate that RA patients with severe inflammation and higher disease activity show lower VPAC_1_ mRNA levels which, in turn, is associated with the predominant proinflammatory Th1 profile^23,26^. In addition, the activation or Th17-differentiation of memory Th lymphocytes from eRA patients increases the mRNA ratio VPAC_2_/VPAC_1_^10,11^. The observed increase in VPAC_2_ expression is greater in EA patients than in HD, which is also detected in other cells involved in this pathology, such as fibroblast-like synoviocytes or macrophage-like synoviocytes^12,14^. The lost in VPAC_1_ mRNA expression together with CD4^+^ T cells activation and VPAC_2_ up-regulation were also observed in other autoimmune diseases such as multiple sclerosis^27^. Besides, in a different immune disease like HIV, while VPAC_1_ protein expression remains unchanged, an increase in VPAC_2_ has been described in Th cells^22^.

Looking beyond the expression pattern, it is also interesting to identify the cellular situation of these receptors. As far as we know, this is the first study to describe that the cellular localization of the VPAC_1_ receptor changes with the activation of Th lymphocytes, which could be important as it might result in other different signaling pathways initiated by VIP in activated T lymphocytes, and may be used as a therapeutic strategy. In resting cells, the receptor is located on both the plasma membrane and inside the cell, in the nucleus, whereas this receptor is mainly restricted to a nuclear location in activated cells. VPAC_2_ receptor is always found in plasma membrane in both resting and activated Th cells. The intracellular position of functional G-Protein Coupled Receptors (GPCRs) in addition to plasma membranes is now broadly accepted by the scientific community^28,29^. In fact, VPAC_1_ receptor presence has been previously described in intracellular compartments such as the nucleus^30,31^. In human glioblastoma cell lines, there is a clear nuclear location for VPAC_1_ receptor, whereas VPAC_2_ receptor has a weak nuclear location^31^. Similar results related to VPAC_1_ receptor were observed in human breast cancer cells, where this receptor is functional in both plasma membrane and nuclear localization. Moreover, its translocation from plasma membrane to nucleus is induced by its own ligand, VIP^30^. In this sense, the palmitoylation of the N-terminal extracellular Cys37 of VPAC_1_ receptor, induced by VIP, mediates the nuclear translocation of this receptor^32^. The traffic of VPAC_1_ receptor from plasma membrane to nucleus could be related to the fact that the sequence of this receptor has a nuclear localization signal sequence in its intracytoplasmic C-terminal, not found in VPAC_2_ receptor^31^. Already in 2006, Goetzl hypothesized that VPAC receptors constitute a dynamic system for signaling in T cells, predicting that responses in the plasma membrane location would have a fast onset and brief duration, whereas receptors in the nuclear membrane would have responses with slow onset and sustained in time^33^. Moreover, some GPCRs such as the metabotropic glutamate receptor 5 are found mainly in the nucleus membrane and remain there via interactions with chromatin, exerting other functions related to transcription, chromosome remodeling and genomic integrity^34^. Thus we cannot rule out the possibility of other different signaling pathways and functions upon the VPAC_1_ receptor activation within the nucleus, which may arise in biological changes of T cells.

Different papers relating to GPCRs intracellular expression prove that these receptors are able to signal intracellularly. For instance, when endocytosed they can continue signaling as they are internalized together with their ligands, but they can also be activated in an autocrine manner, as ligands are synthesized *in situ*. Moreover, it has been described that some administrated ligands co-localize with their cognate receptors in the nucleus and are able to activate the downstream signaling pathways, speculating that ligands could be transported intracellularly using some active uptake mechanism^35^. More pathways can be used by the ligands to enter inside the cell and/or inside the nuclear envelope, such as endocytosis, channels or pores. Moreover, although VPAC_1_ is less expressed at mRNA level on activated T cells, its protein expression remains invariable; therefore, it is probably signaling from the inside.

Taking into account these facts, redistribution of GPCRs in cells changes the spatial and temporal compartmentalization of cAMP and could have differential functional actions on T cells^36^. cAMP has distinct effects on T cells, since it acts as a positive regulator of physiological T cell functions, whereas a short burst of cAMP induced by TCR is required for T cell activation. On the contrary, a sustained elevation in intracellular cAMP also acts as an immunosupressor. These differential effects of cAMP can be explained by different factors including the amount of cAMP produced, the cAMP effectors involved, and the spatial and temporal compartmentalization of cAMP^37^. Our results point to an upregulation of cAMP levels induced by VIP receptors in both resting and activated T cells. Once inside the cell, cAMP triggers various downstream pathways, mainly the canonical PKA pathway and the non-canonical EPAC pathway. Depending on the cell type, EPAC and PKA may act independently or synergistically or oppose to each other in regulating specific cellular functions^38^. In Th cells, the effects of the rise in cAMP, required for their activation, are mainly mediated by PKA whereas a sustained elevation of cAMP, necessary to activate EPAC1, leads to suppression of T cell activation, proliferation and chemotaxis^37,39,40^. Our results indicate that both receptors signal through cAMP-PKA-CREB pathway in resting cells, whereas the main signaling pathway in activated Th cells is cAMP-EPAC-Rap1.

The majority of studies related to the involvement of each VPAC receptor subtype on the immunomodulatory role of VIP, have been performed using receptor agonists and antagonists in different animal models, or VPAC-deficient mice. Although VPAC_1_ has been the main receptor involved in VIP action, more nuanced views suggest the participation of VPAC_2_ in immune regulation^1,3,41,42^. Focusing on human CD4^+^ T cells, there are few *in vitro* studies addressing this issue. During Th17 polarization from human naïve Th cells, VIP maintains a non-pathogenic profile through up-regulation of RORC, RORA, IL-17, IL-23R or STAT3. VPAC_1_ and VPAC_2_ are responsible for modulating the first three molecules; meanwhile VIP exerts upregulation of IL-23R through VPAC_2_ receptor and upregulation of STAT3 through VPAC_1_ receptor^9^. The pathogenic Th profile of human memory Th cells, after seven days of activation, is downregulated by VIP in HD and eRA patients, decreasing T-bet, STAT3, IL-2, IL-22, IL-12Rβ2, IL-23R or GM-CSF expression^10^. Our results indicate that all these molecules peak at day 1 of the Th cell activation, and VIP downregulates the abovementioned molecules through both VPAC receptors. Thus, the pathogenic profile of these cells can be modulated by both VIP receptors in our system.

Among the different activation markers of Th cell tested, we only found an up-modulatory effect of VIP on CD30 expression after four days of activation. CD30 antigen is a member of the TNF receptor superfamily that is expressed by activated memory Th cells but not by resting T cells. A soluble form of CD30 is released by CD30^+^ cells *in vitro* and *in vivo*, being sCD30 level closely related to cell CD30 expression^43^. CD30 induced signals mediated by NFκB and TRAF molecules cause cell proliferation or cell death. In addition several studies point to a role of sCD30 during Th differentiation and in the regulation of memory Th cell response^44,45^. CD30, as well as its soluble form sCD30, is an important costimulatory molecule in the regulation of the balance between Th1 and Th2 response^43,44^. Although *in vitro* studies have mainly associated the CD30 expression with a Th2/Th0 phenotype, *in vivo* studies suggest that CD30/CD30L signaling is involved in Th2 responses and Th2- associated diseases but also in Th1 and Th17 responses or pathologies such as RA^44,45^. In fact, CD30 T cells are proposed to exert an anti-inflammatory activity in RA. It has been described a high percentage of Treg cells in the synovial fluid of RA patients, presumably in order to control and block the disease progression. Almost 50% of these cells are expressing CD30, which enhances the idea that CD30 is expressed by cells with anti-inflammatory profiles^45,46^. As VIP is able to upregulate the expression of this marker in activated T cells, it may serve as an immunomodulatory agent. The mechanism by which VIP is able to upregulate CD30 and sCD30 expression could be by diminishing the IFNγ/IL4 ratio observed in activated T cells *in vitro*^8^ and in peripheral blood lymphocytes after polyclonal activation^6^, since it has been described that the expression of CD30 is controlled by the balance between IL-4 and IFNγ^47^. Moreover, VIP induce Th2 differentiation and a non-pathogenic Th17 polarization in human Th cells, which may explain the VIP effects observed on CD30 expression^1,3,9,11^.

An additional important characteristic of Th lymphocytes is their capacity to migrate from the bloodstream to inflamed tissues, where the expression pattern of chemokine receptors turns into essential to carry out this purpose. We tested CXCR4 and CCR7 as chemokine receptors that appear in naïve Th cells and could be modulated during Th activation and differentiation. CCR6 and CXCR3 where studied as two representative chemokine receptors linked to pathogenicity of Th cells, Th17 and Th1 subtypes^48,49^. Whereas the surface expression of CXCR4 and CCR7 were increased at day 1 after cell activation, CCR6 and CXCR3 were down-regulated. Chemokines and their receptors act in complex networks. Chemokine receptors form homo- and heterodimers, as well as higher order structures at the cell surface, in all cases dynamic structures^50^. Thus, we also checked the capacity of migration of the cells. This ability only increased in activated Th cells towards CCL19 and CXCL10, the ligands for CCR7 and CXCR3, respectively. That means that the activation of memory Th cells increases the chemokine receptors necessary for their homing and this is related to a Th1 profile. The presence of VIP or either VPAC agonist during the activation maintains the surface expression of all chemokine receptors studied and their functional migration capacity *vs* CCL19, CXCL12, CCL20 and CXCL10. Previous *in vivo* and *in vitro* studies have demonstrated that VIP down-regulates CXCL10/CXCR3, CXCL12/CXCR4, CCL20/CCR6, CCL19/CCR7 axis in different immune cells^51–53^. In other cases, VIP increases CCR6 surface expression during Th17 differentiation or polarization of Th cells towards a non-pathogenic phenotype^9,10^.

To sum up, this report dissects for the first time the role of VIP receptors, VPAC_1_ and VPAC_2_, during the activation of human memory Th cells. Both receptors exhibit high capacity of immunomodulation controlling the pathogenic profile, the activation markers and the migration ability. These results highlight the expression pattern and cellular localization of VPAC receptors, as well as their signaling pathways involved during the activation of Th cells. The change in VPAC expression pattern during the activation of memory Th cells is similar in HD and EA patients, which means that both receptors have immunomodulatory effects also in EA patients. The dissection of the behavior of VIP receptors can contribute to their use either as markers of activity in autoimmune diseases or to the development of new therapies based on their blocking and/or activation.

## Methods

### Healthy donors

Samples from 16 Healthy Donors (HD) were included in this study. The study was performed according to the recommendations of the Declaration of Helsinki and was approved by the Ethics Committees of the Transfusion Center of CAM. HD samples were obtained from buffy coats from the Transfusion Center. Following the Spanish Personal Data Protection law, the patients’ demographic information was confidential. All patients signed an informed consent form before sampling.

### Patients

Samples from 14 patients on an EA register were analyzed (57% RA, 43% undifferentiated arthritis, and median age 49 years; mean disease duration at entry, 3.2 months). All patients belongs to PEARL’s cohort from Hospital de la Princesa, approved by Ethics Committees of La Princesa Hospital (Madrid, Spain) (PI-518). All patients signed an informed consent form before data were included in the register, and biological samples were stored at the local Biobank. Only data from patients fulfilling the 2010 American College of Rheumatology/European League Against Rheumatism criteria for RA within the 5-year follow-up (n = 7) or with chronic undifferentiated arthritis (n = 9) were analyzed^54^. Blood samples were collected before treatment prescription.

### Isolation of human peripheral blood memory T cells

Memory Th cells were isolated from whole blood from HD or eRA patients. For mononuclear cell isolation, density gradient centrifugation by Ficoll–Hypaque (Sigma Aldrich, St Louis, MO, USA) was done. CD4^+^ T cells were isolated by negative selection using a CD4^+^ T Cell Isolation Kit II (Miltenyi Biotec, San Diego, CA, USA). CD4^+^CD45RO^+^ T cells were then isolated by negative selection using CD45RA^+^ MicroBeads (Miltenyi Biotec). The purity of CD4^+^CD45RO^+^ T cells was greater than 92%.

### *In vitro* expansion of human memory T cells

CD4^+^CD45RO^+^ T cells were cultured in 96 U-well plates (0.1 × 10^6^ cells/well) with RPMI-1640-GlutaMAX media (Life Technologies, Carlsbad, CA, USA) supplemented with 10% FBS (Lonza, Basel, Switzerland) and 1% penicillin/streptomycin (Life Technologies). Cells were activated/expanded with anti-CD3/anti-CD28 coated beads (Life Technologies). CD4^+^CD45RO^+^ T cells were cultured in the absence (activation condition) or presence of 10 nM of VIP (Polypeptide group, Strasbourg, France), VPAC_1_ agonist (K^52^R^1,6^L^2,7^VIP^53,54^ (1–7)/GRF (8–27)) or VPAC_2_ agonist (RO 25–1553) (Bachem A.G., Bubendorf, Switzerland) at different days (1, 4, 7). Cells without any treatment or activation and recollected at day 0 were considered resting cells.

### Isolation of plasma membrane and nuclear fractions

Resting (day 0) and activated (day 7) cells were washed with ice-cold PBS and lysed by 40 strokes in 10 mM Tris-HCl (pH 7.4) containing 10 mM NaCl, 3 mM MgCl_2_, 10 µg/ml aprotinin, 10 µg/ml leupeptin, 10 µg/ml pepstatin, 2 mM PMSF and 1 mM Na_3_VO_4_. The homogenates were centrifuged at 800 × g for 20 min at 4 °C. The supernatant was centrifuged at 100,000 × g for 20 min at 4 °C and the pellet, corresponding to the plasma membranes, was resuspended in radioimmunoprecipitation assay (RIPA) buffer (50 mM Tris-HCl, ph 7.5, 150 mM NaCl, 30 mM NaF, 5 mM EDTA, 1% Triton X-100, 1% Nonidet P-40, 0,1% SDS, 1 mM DTT, 1 mM orthovanadate, proteinase inhibitor cocktail) and stored at −80 °C until use. The pellet from the first centrifugation was resuspended in 10 mM Tris-HCl (pH 7.4) containing 1.5 mM MgCl_2_, 10 mM KCl, 2 mM DTT, 10 µg/ml aprotinin, 10 µg/ml leupeptin, 10 µg/ml pepstatin, 2 mM PMSF and 1 mM Na_3_VO_4_, incubated on ice for 10 min and centrifuged at 17,000 × g for 10 min at 4 °C. The supernatant was discarded and the pellet was resuspended in 20 mM Tris–HCl (pH 7.4) containing 0.5 M NaCl, 20% glycerol, 1.5 mM MgCl_2_, and 10 µg/ml aprotinin, 10 µg/ml leupeptin, 10 µg/ml pepstatin, 2 mM PMSF and 1 mM Na_3_VO_4_. After incubation on ice for 30 min and centrifugation at 20,000 × g for 20 min at 4 °C, the resulting supernatant corresponded to the nuclear fraction and was stored at −80 °C until use.

### RNA extraction and semiquantitative real-time PCR

For total RNA extraction, we used TriReagent method (Sigma Aldrich). RNA (2 µg) were reverse transcribed using a High Capacity cDNA Reverse Transcription Kit (Life Technologies). Semiquantitative RT-PCR analysis for all molecules tested (Table 2) was performed using TaqMan Gene Expression Master Mix (Applied Biosystems, Waltham, MA, USA). We normalized each sample with β-actin, using the formula 2^−ΔCt^. Amplification was performed in a 7900HT Fast Real-Time PCR System apparatus (Applied Biosystems).

### Immunocytochemistry staining

Cell suspensions (resting- and activated-cells at day 7) were seeded in PBS 1x and hold onto SuperFrost Plus slides (Thermofisher Scientific) 30 minutes at 37 °C, 5%CO2. Then slides were washed with PBS 1x and fixed. After rehydration and blocking, slides were incubated with rabbit polyclonal anti-human VPAC_1_ antibody (1:100, Thermo Fisher Scientific, Paisley, UK) and mouse monoclonal anti-human VPAC_2_ (1:50, Abnova) 1 hour at RT. After washing, Alexa Fluor 488 donkey anti-rabbit IgG and Alexa Fluor 594 goat anti-mouse IgG (1:500, Life Technologies) were used as secondary antibodies (1 hour at RT). Counterstaining was performed with 1 mg/ml Hoechst. Negative controls were performed in the absence of anti-VPAC_1_ and anti-VPAC_2_ antibodies. Fluorescence was examined on a Leica SP-2 Acousto-Optical Beam Splitter confocal microscope with inverted stand (Leica DM IRE2; objective, 63X; Leica, St. Gallen, Switzerland). Images were analyzed by ImageJ (Fiji).

### Western blot

Protein extracts were obtained in ice-cold RIPA buffer. Protein extracts (10 and 40 µg for VPAC_1_ and VPAC_2_, respectively) were subjected to sodium dodecyl sulfate polyacrylamide gel electrophoresis (SDS-PAGE) and blotted on a polyvinylidene difluoride (PVDF) membrane (Bio-Rad Laboratories, France). After blocking, membranes were incubated overnight at 4 °C with rabbit polyclonal anti-human VPAC_1_ antibody (1:10000, Thermo Fisher Scientific) and mouse monoclonal anti-human VPAC_2_ antibody (1:1000, Abnova, Tapei, Taiwan). Mouse monoclonal anti-beta actin (ACTB) (1:10.000, Sigma Aldrich) was used as loading control. Appropriate horseradish peroxidase-conjugated secondary antibodies (1:10.000, Santa Cruz Biotechnology, Dallas, TX, USA) were applied and detected by Pierce SuperSignal West Pico (Thermo Fisher Scientific). Protein bands were analyzed using the Bio-Rad Quantity One program and normalized against β-actin.

Membrane and nuclear fraction extracts from resting and activated cells were subjected to SDS-PAGE and blotted on a nitrocellulose membrane (Bio-Rad). VPAC_1_ and VPAC_2_ antibodies were used as above mentioned. Rabbit anti-human Na^+^/K^+^ ATPase (1:1000, Cell Signaling Technology, Leiden, The Netherlands) was used as plasma membrane marker. Appropriate horseradish peroxidase-conjugated secondary antibodies (1:5000, Cell Signaling and 1:10000 Santa Cruz, respectively) were applied and detected by LumiGLO® (Cell Signaling). Protein bands were analyzed using with the Bio-Rad Quantity One program and normalized against fraction markers.

To detect cyclic AMP response element binding (CREB) phosphorylation, memory Th cells were cultured 2 hours without any stimuli (resting) or 7 days with anti-CD3/anti-CD28 coated beads (activated). In both conditions, cells were cultured in serum-free medium 30 min before adding VIP, VPAC_1_ or VPAC_2_ agonist for 15 minutes. Cells were washed in ice-cold tris-buffered saline (TBS) and lysed in RIPA buffer. After centrifugation, supernatants were quantified and frozen until western blot was performed. Protein extracts (10 µg) were subjected to SDS-PAGE and blotted on a PVDF membrane (Bio-Rad). After blocking, membranes were incubated overnight at 4 °C with rabbit anti-human pCREB antibody and rabbit anti-human CREB antibody (both 1:1000, Cell Signaling Technology) using the later as loading control. Horseradish peroxidase-conjugated anti-rabbit antibody (1:5000, Cell Signaling) was applied and detected by LumiGLO® (Cell Signaling). Protein bands were analyzed using the Bio-Rad Quantity One program and normalized against total CREB.

### Pull down assays for detection of activated Rap1

To quantify activated Rap1, we performed active Rap1 pull-down assays (Thermo Scientific) following the manufacturer’s instructions. In brief, memory Th cells were cultured 7 days with anti-CD3/anti-CD28 coated beads (activated), and then 15 minutes in presence or absence of VIP or VPAC_1_ and VPAC_2_ agonists. After treatment, cells were washed with ice-cold TBS and lysed in lysis buffer. Positive control was obtained by treatment of lysates with GTPγS for 30 minutes. Cell lysates were incubated with a Rap1 binding domain-GST fusion protein and subjected to a glutathione-agarose resin, resulting in precipitation of activated GTP bond Rap1. The precipitates were subjected to standard SDS-PAGE and blotted on a nitrocellulose membrane (Bio-Rad). Primary and secondary antibodies from the manufacturer kit were used and detected by Pierce SuperSignal West Pico (Thermo Fisher Scientific). Protein bands were analyzed using the Bio-Rad Quantity One program.

### Measurement of cytokines

Levels of Granolocyte-Macrophage Colony Stimulation Factor (GM-CSF) and soluble CD-30 (sCD30) were measured from cell culture supernatants by Enzyme-Linked Immuno Sorbent Assay (ELISA) test (eBioscience, San Diego, CA, USA), according to the manufacturer’s instructions.

### Measurements of cAMP levels

Intracelullar cyclic adenosilmonophosphate (cAMP) levels were measured after 1 hour of stimulation with 10 nM of VIP or VPAC agonists by Enzyme-Linked Immuno Sorbent Assay (ELISA) test (Enzo Life Sciences, NY, USA) from cell lysate (0,1 M HCL solution), according to the manufacturer’s instructions. cAMP levels were corrected by protein content of cell lysate measured by QuantiPro^TM^ BCA Assay Kit (QBCA).

### Flow cytometry analysis

After one day of culture, cells were collected and labeled with APC-conjugated CD30 (clone BerH8), PE-conjugated CCR6 (clone 11A9), FITC-conjugated CCR7 (Clone 150503), APC-conjugated CXCR3 (clone 1C61) or PE-Cy5-conjugated CXCR4 (clone 12G5), all from BD Biosciences. Autofluorescence and isotype controls were set up to define non-specific fluorescence. Cytometric analysis was performed using a BD FACScalibur flow cytometer (BD Biosciences, using BD FACSDiva software). Data analysis was performed using FCS Express v3 (De Novo Software, Glendale, CA, USA).

### Cell migration assay

Cell migration was evaluated using 6.5 mm Transwell plates with 5.0 µm Pore Polycarbonate Membrane Insert (NY, USA). 300.000 cells (100 µl), previously resuspended in chemotaxis medium (RPMI 1640, 0.5% FBS, 10 Mm HEPES), were placed in the upper chamber of the Transwell. Different concentrations of CXCL12 (20 nM), CXCL10 (50 nM), CCL19 (100 nM and 50 nM) and CCL20 (100 nM and 50 nM) in chemotaxis medium was added to lower chamber of Transwell. Chemotaxis medium without stimuli was added to the lower chamber as a control. Plates were incubated 2 hours at 37 °C, 5% CO_2_. Cells which had migrated to the lower chamber were counted in a EPICS XL flow cytometer (Beckman Coulter), and expressed as a percentage of input cells

### Statistical analysis

Parametric test (*t* test and one way-ANOVA) were used to compare different cell populations. Statistical tests were done using GraphPad Prism Version 6.0 software (GradphPad Software).

## Supplementary information

Supplementary-Original Western Blot
