# Supplementary material for: Activation of Th lymphocytes alters pattern expression and cellular location of VIP receptors in healthy donors and early arthritis patients
Source: Sci Rep. 2019 May 14;9:7383. doi: 10.1038/s41598-019-43717-2 (PMC6517580; doi:10.1038/s41598-019-43717-2)

SUPPLEMENTARY FILE: ORIGINAL WESTERN BLOTS FROM FIGURES 1, 2, 4 AND 8.

Activation of Th lymphocytes alters pattern expression and cellular location of VIP receptors in healthy donors and early arthritis patients

Villanueva-Romero R, Gutiérrez-Cañas I, Carrión M, González-Álvaro I, Rodríguez-Frade J M, Mellado M, Martínez C, Gomariz R P and Juarranz Y

Western Blot from Figure_1

Western Blot $VPAC_1$

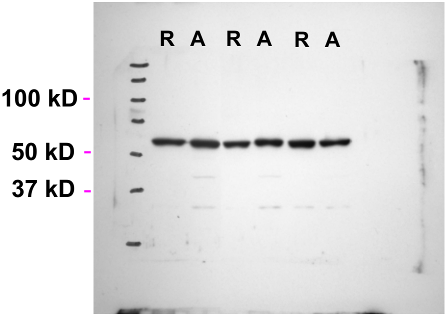

Western Blot $VPAC_2$

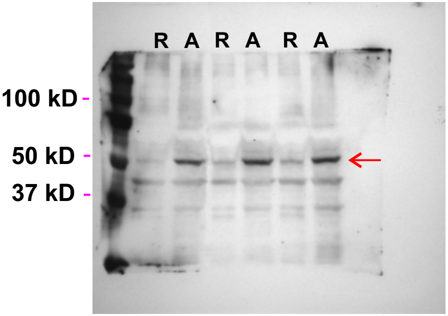

ß-actin WB $VPAC_1$

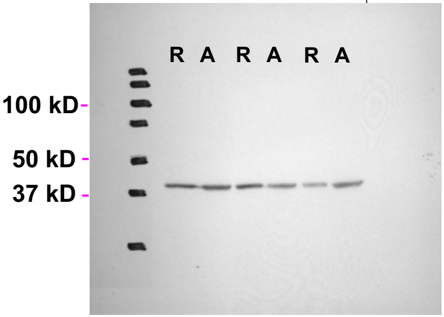

ß-actin WB $VPAC_2$

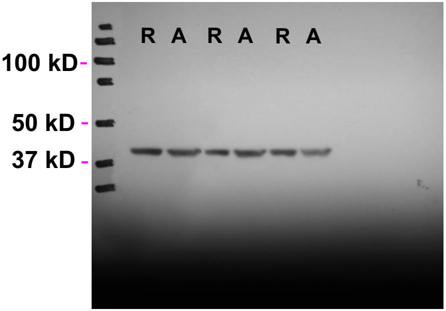

Western Blot from Figure_2

Resting Th cells

Western Blot VPAC$_1$

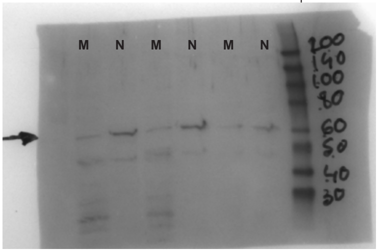

Western Blot VPAC$_2$

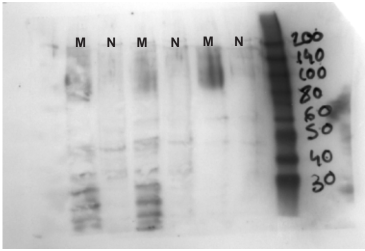

Western Blot Na^+/K^+ ATPase

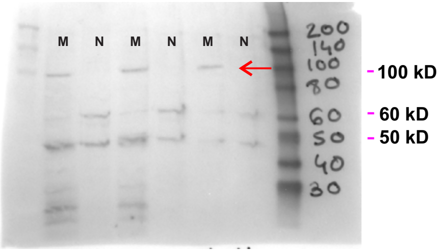

Activated Th cells

Western Blot VPAC$_1$

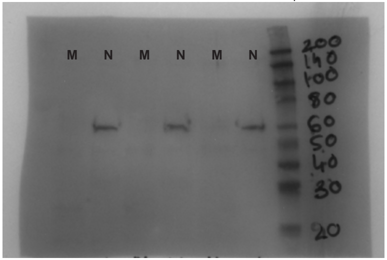

Western Blot VPAC$_2$

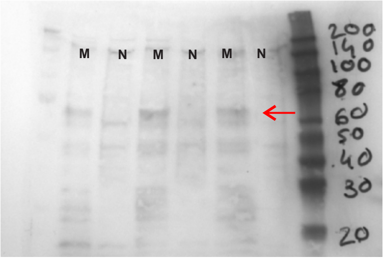

Western Blot Na^+/K^+ ATPase

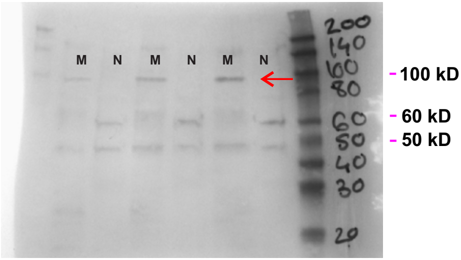

Western Blot from Figure_4

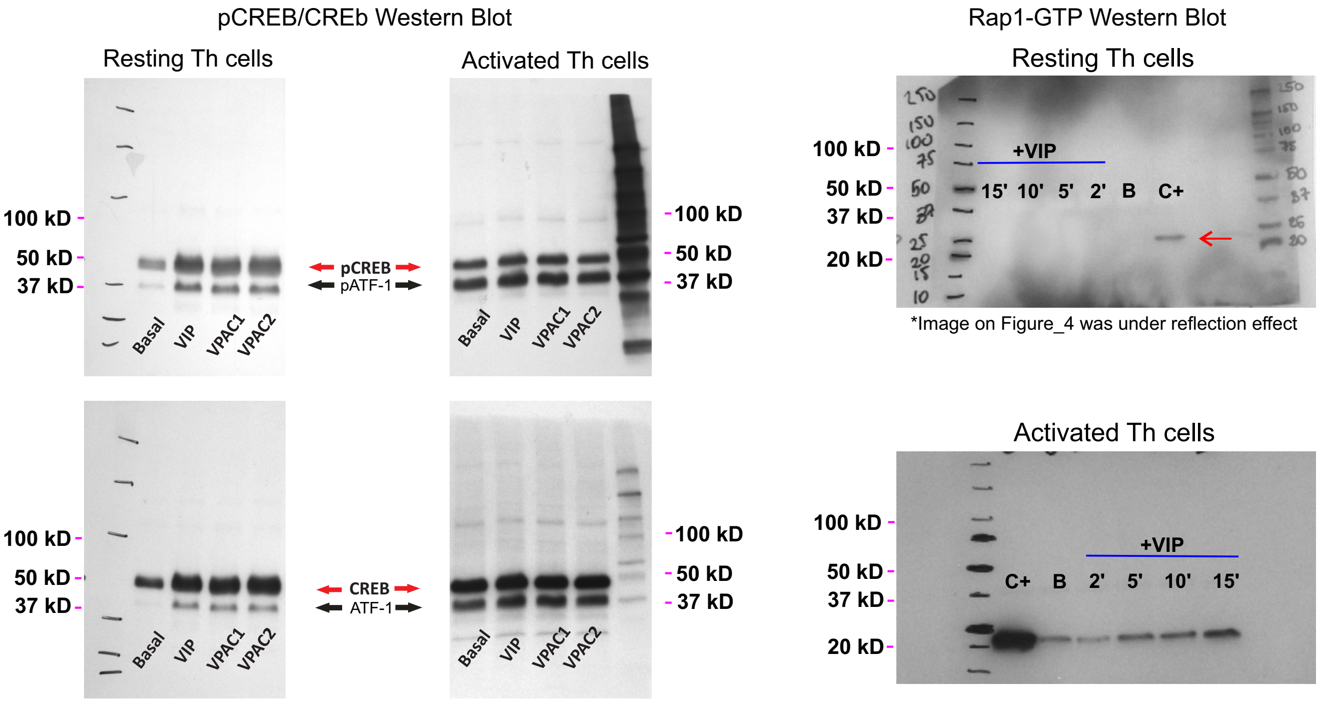

Western Blot from Figure_8

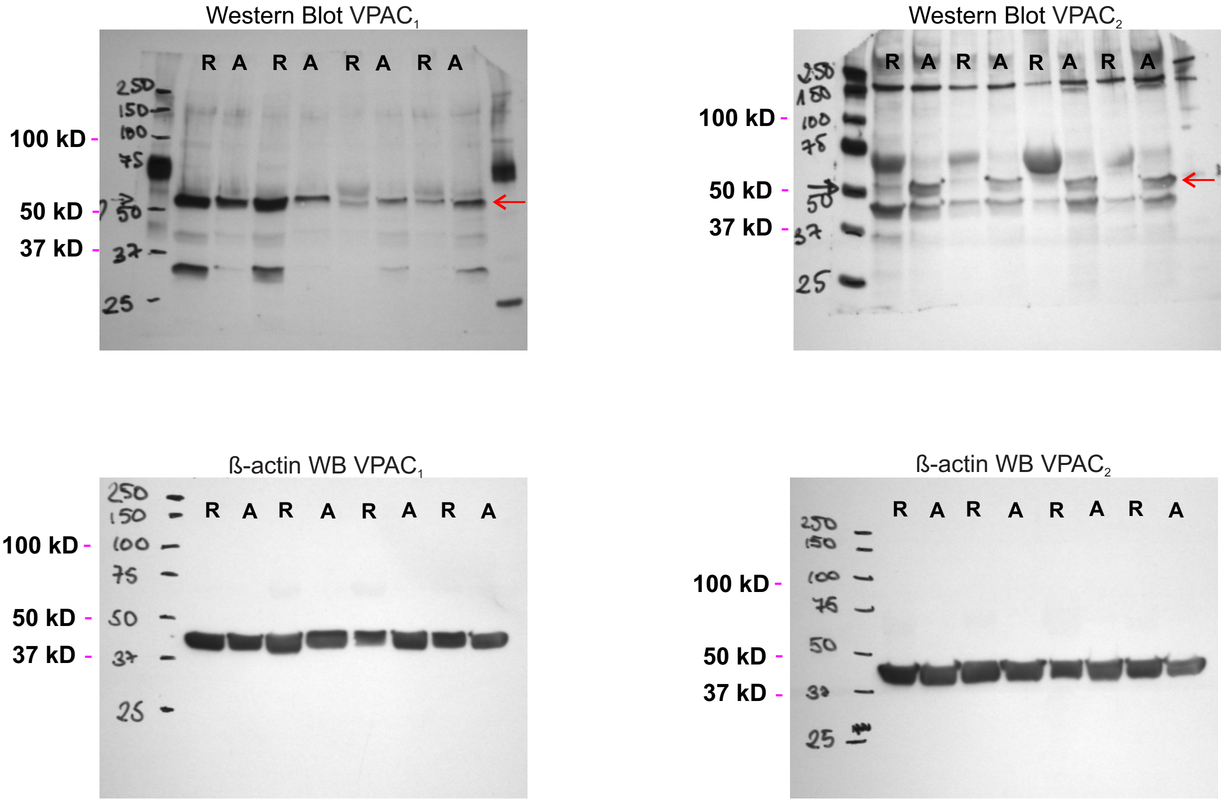

Supplement: Supplementary file 1 — Supplementary-Original Western Blot [file 41598_2019_43717_MOESM1_ESM.pdf]
